# 

**Published:** 1863-07

**Authors:** 


### WORKS PUBLISHED DURING THE QUARTER.

### 1. *Medicine.*

Barker.—On Malaria and Miasmata, and their influence in the production of Typhus and Typhoid Fevers, Cholera, and the Exanthemata: founded on the Fothergillian Prize Essay for 1859. By Thomas Herbert Barker, M.D., F.R.S., Edin. 8vo, cloth, 8s.

Beale.—The Stomach Medically and Morally Considered: Lectures delivered at the St. Martin's Library Reading-room. By Lionel J. Beale, M.R.C.S. Fcap. 8vo, sewed 1s.

Davy.—Physiological Researches. By John Davy, M.D., F.R.S. 8vo, cloth. 15s.

Epidemiological Society.—Transactions of the Epidemiological Society of London. Part III. (completing the First Volume.) 8vo, sewed, 5s.

Fuller.—Diseases of the Heart and Great Vessels: their Pathology, Physical Diagnosis, Symptoms, and Treatment. By H. W. Fuller, M.D. Cantab., F.R.C.P. Lond. 8vo, cloth, 7s. 6d.

Graves.—Studies in Physiology and Medicine. By Robert James Graves, M.D., F.R.S. Edited by William Stokes, M.D. With Portrait and Memoir. 8vo, cloth, 14s.

Harley.—Jaundice: its Pathology and Treatment. With the Application of Physiological Chemistry to the Detection and Treatment of Diseases of the Liver and Pancreas. By George Harley, M.D. With Illustrations. 8vo, cloth, 7s. 6d.

Hassall.—The Urine in Health and Disease: being an Exposition of the Composition of the Urine, and of the Pathology and Treatment of Urinary and Renal Disorders. Second Edition, considerably enlarged, with 79 Engravings (23 coloured). By A. H. Hassall, M.D. 8vo, cloth, 12s. 6d.

Leared.—Imperfect Digestion: its Causes and Treatment. By Arthur Leared, M.D. Third Edition. Fcap. 8vo, cloth, 4s.

Lee.—The Baths of Germany. By Edwin Lee, M.D. Fourth Edition. Post 8vo, cloth, 7s.

Lee.—The Baths of Nassau. By Edwin Lee, M.D. Post 8vo, 2s. 6d.

---

Obstetrical Society.—Transactions of the Obstetrical Society of London, Vol. IV., for the year 1862. With a List of Officers, Fellows, &c. With Plates. 8vo, 15s.

Routh.—Infant Feeding, and its Influence on Life; or, the Causes and Prevention of Infant Mortality. By Charles H. Routh, M.D. Second Edition. Fcap. 8vo, cloth, 6s.

Smith.—On Human Entozoa; comprising the Description of the Different Species of Worms found in the Intestines and other Parts of the Human Body, and the Pathology and Treatment of the various Affections produced by their presence. To which is added a Glossary of the Principal Terms employed. By W. Abbots Smith, M.D. 8vo, 6s.

Snellon.—Test-Types for the Determination of the Acuteness of Vision. By H. Snellon. 8vo, 4s.

Squire.—The Pharmacopœias of Thirteen of the London Hospitals, arranged in Groups for Easy Reference and Comparison. By Peter Squire. 16mo, cloth, 3s. 6d.

Squire —On Pepsine. By M. Boudault. Translated by W. Stevens Squire, Ph.D., F.C.S. Third Edition. 6d.

Townley.—Parturition Without Pain or Loss of Consciousness. By James Townley, M.R.C.P., Edin. Third Edition. Post 8vo, cloth, 2s. 6d.

### 2. *Surgery.*

Ashton.—On the Diseases, Injuries, and Malformations of the Rectum and Anus. By T. J. Ashton. With Engravings. Fourth Edition, 8vo, cloth, 8s.

Barwell.—On the Cure of Club-foot without Cutting Tendons, and on Certain New Methods of Treating other Deformities. By Richard Barwell, F.R.C.S. With Seventeen Engravings, fcap. 8vo, cloth, 3s. 6d.

Clark.—Outlines of Surgery: being an Epitome of the Lectures on the Principles and Practice of Surgery delivered at St. Thomas's Hospital. By F. Le Gros Clark, F.R.C.S. Fcap. 8vo, cloth, 5s.

Haughton.—Outlines of a New Theory of Muscular Action. By the Rev. Samuel Haughton, M.D. Crown 8vo, 1s. 6d.

Hogg.—A Manual of Ophthalmoscopic Surgery: being a Practical Treatise on the Use of the Ophthalmoscope in Diseases of the Eye. By Jabez Hogg. With Coloured Plates. Third Edition, 8vo, cloth, 10s. 6d.

Hunt.—Guide to the Treatment of the Diseases of the Skin; with Suggestions for their Prevention. For the Use of the Student and General Practitioner. Illustrated by Cases. By Thomas Hunt, F.R.C.S. Seventh Edition. Fcap. 8vo, cloth, sewed, 2s. 6d.

Thompson.—Practical Lithotomy and Lithotrity; or, an Inquiry into the Best Modes of Removing Stone from the Bladder. By Henry Thompson, F.R.C.S. With numerous Engravings, 8vo, cloth, 9s.

Wood.—On Rupture, Inguinal, Crural, and Umbilical; the Anatomy, Pathology, Diagnosis, Cause, and Prevention; with New Methods of effecting a Radical and Permanent Cure; embodying the Jacksonian Prize Essay of the Royal College of Surgeons, London, for 1861. By John Wood, F.R.C.S. Eng. With numerous Illustrations by Bags. 8vo, cloth, 2s. 6d.

### 3. *Chemistry.*

Buckmaster.—Elements of Chemistry, Inorganic and Organic. By J. C. Buckmaster. Second Edition, enlarged. 18mo, cloth, sewed, 3s.

Noad.—Manual of Chemical Analysis, Qualitative and Quantitative. For the Use of Students. Part I., Qualitative. By Henry M. Noad, Ph. D., F.R.S., F.C.S. Crown 8vo, cloth, 6s.

Kemp.—A Description of certain Dry Processes in Photography, specially adapted to the Use of the Tourist; with Supplementary Notice of Plans useful to the Scientific Traveller and Missionary. By George Kemp, M.D. Small 8vo, sewed, 2s.

---

### FOREIGN MEDICO-PSYCHOLOGICAL LITERATURE.

